# Flow and intuition: a systems neuroscience comparison

**DOI:** 10.1093/nc/niae040

**Published:** 2025-01-04

**Authors:** Steven Kotler, Darius Parvizi-Wayne, Michael Mannino, Karl Friston

**Affiliations:** Flow Research Collective, Gardnerville, Nevada, USA; Department of Philosophy, Macquarie University, Sydney, New South Wales, Australia; Flow Research Collective, Gardnerville, Nevada, USA; Artifical Intelligence Center, Miami Dade College, Miami, Florida, USA; VERSES AI Research Lab, Los Angeles, CA, United States; Queen Square Institute of Neurology, University College London, London, United Kingdom

**Keywords:** flow, intuition, active inference, consciousness, dual-processing, habits

## Abstract

This paper explores the relationship between intuition and flow from a neurodynamics perspective. Flow and intuition represent two cognitive phenomena rooted in nonconscious information processing; however, there are clear differences in both their phenomenal characteristics and, more broadly, their contribution to action and cognition. We propose, extrapolating from dual processing theory, that intuition serves as a rapid, nonconscious decision-making process, while flow facilitates this process in action, achieving optimal cognitive control and performance without [conscious] deliberation. By exploring these points of convergence between flow and intuition, we also attempt to reconcile the apparent paradox of the presence of enhanced intuition in flow, which is also a state of heightened cognitive control. To do so, we utilize a revised dual-processing framework, which allows us to productively align and differentiate flow and intuition (including intuition in flow). Furthermore, we draw on recent work examining flow from an active inference perspective. Our account not only heightens understanding of human cognition and consciousness, but also raises new questions for future research, aiming to deepen our comprehension of how flow and intuition can be harnessed to elevate human performance and wellbeing.

## Introduction

Intuition and flow are two related, yet distinct, cognitive phenomena that both involve the processing of information. In the language of neural dynamics—and for the purposes of this paper—‘information processing’ will be defined as the process by which organisms take in information about the world through sensory organs, learn statistical regularities about that information, and use those regularities to navigate and interact with the environment (Varley et al. 2023). As we will discuss below, according to the active inference framework this can be cast as a reduction of uncertainty or, more technically, surprisal given a generative model (Friston 2010, Friston et al. 2016, Kirchhoff et al. 2018). When you have an intuition about a possible next action—for example, the familiar gut sense of ‘don’t walk down that alley’—your intuition is guiding you away from unpredicted observations (uncertainty) and towards those observations that you—because of the thing that you are—prefer. From the perspective of active inference, those preferred observations *just are* the predictions (or priors) that make up the agent’s generative model (i.e. probabilistic expectations; Smith et al. 2022). In flow, which can be defined as ‘an optimal state of consciousness’ (Csikszentmihalyi 2000), rapid flows of skilful action are deployed to achieve the sensory outcomes the agent prefers. In other words, flow, like intuition, subtends the requisite minimization of uncertainty (cf., free energy) (Parvizi-Wayne et al. 2024).

Before discussing these technical points further, it is worth offering a brief introduction to what flow and intuition are. A flow state is an ‘almost automatic, effortless, yet highly focused state of consciousness’ (Csikszentmihalyi 1997, p. 110) that defines the experience of a skilled actor exercising their expertise within a sufficiently challenging task (Nakamura and Csikszentmihalyi 2009, 2014). Phenomenologically, one of the hallmarks of flow is a sense of always knowing what to do next, which is described formally as a ‘sense of control’ or expertise.

Neurophysiologically, this is underwritten by the activation of neural networks associated with cognitive control and goal-directedness (Huskey et al. 2018, 2022, Kotler et al. 2022). This sense-knowing appears within the action-perception cycle, as an embodied signal that co-arises with the behaviour itself. In other words, we know what to do and are already doing it by the time we realize that we know—which is what psychologists mean by ‘the merger of action and awareness’, another of flow’s hallmark experiential characteristics (Gold and Ciorciari 2020, Kotler et al. 2022, Shepherd 2022). This points to flow as a particularly noteworthy state of consciousness, which is characterized by, among other things, total immersion in a challenging task, a distortion of time perception, and an elimination of reflective self-awareness (Csikszentmihalyi 1990, 1997, Nakamura and Csikszentmihalyi 2009, 2014, Christandl et al. 2018, Abuhamdeh 2020, Parvizi-Wayne 2024, Parvizi-Wayne et al. 2024).

Intuition, conversely, is a form of nonconscious cognition ‘resulting in a spontaneous tendency toward a hunch or hypothesis’ (Zander et al. 2016, p. 2; cf., Fridland and Stichter 2021, Hogarth 2001, Lufityanto et al. 2016, Topolinski and Strack 2008). Furthermore, intuitions are based on implicit or tacit knowledge acquired throughout a lifetime (Bowers et al. 1990). Finally, they often manifest as *affective* tendencies towards action—i.e. a *felt* go or no-go signal strong enough to draw the individual into action (Gigerenzer 2008), and can thus be contrasted with insights, which Zander et al. (2016) (p. 3) describe as ‘the *sudden and unexpected understanding* of a previously incomprehensible problem or concept’ (italics original). Note that insights, while sharing characteristics with intuitions, often involve a more dramatic cognitive shift and, interestingly, tend to be more accurate than analytic solutions (Salvi et al. 2016). From an active inference perspective, insights could be conceptualized as sudden, large reductions in the uncertainty entailed by the likelihood mappings. This aligns with the subjective experience of certainty and clarity often reported during insight moments (Laukkonen et al. 2023). Unfortunately, a further discussion concerning the difference between insight and intuition, and their description within the active inference framework, is beyond the scope of this paper (cf., Zander et al. 2016, Friston et al. 2017, Laukkonen et al. 2023).

We propose that flow relies upon many aspects of intuitive decision-making, including a sense of ‘knowing’ what to do without conscious (declarative) analysis. Kotler (2006, 2014) reported anecdotally that subjects in flow report intuition being clearer and more accurate than normal, but this idea has neither been formally tested nor empirically explained. Little research has explored the relationship between flow and intuition, especially at the neurobiological level. That said, Järvilehto (2016, p. 96) has examined the question psychologically and pointed out that ‘both flow and intuition concern our capacity for cognition and action without heavy input from reflective and conscious cognitive mechanisms’. He posits that flow is intuition in action, and intuition is cognition in flow. Furthermore, within the context of dual processing theory (DPT), Järvilehto (2016) argues that both intuition and flow involve so-called system 1 cognitive processing, which is typically described as fast, non-conscious and parallel, differing from so-called system 2 cognitive processing, which is typically described as slow, conscious and serial (Sloman 1996, Evans 2003, Kahneman 2011, Da Silva 2023). In this paper, we will address these claims, attempting to extend them into cognitive neurodynamics, where, in recent years, researchers have begun to explore the neurobiology of both flow and intuition (Volz and von Cramon 2006, Volz et al. 2008, Klasen et al. 2012, de Manzano et al. 2013, Horr et al. 2014, Ulrich et al. 2014, Lufityanto et al. 2016, Zander et al. 2016, Huskey et al. 2018). However, no one has yet compared these data nor attempted to unravel the relationship between these two processes. This leaves many interesting open research questions. For example, as intuitive processes appear to be active in the state of flow, can nonconscious information processing be governed by cognitive control mechanisms? In other words, if flow is intuition in action, as Järvilehto (2016) purports, it is unclear how intuition as nonconscious processing and flow as a state of high cognitive control can be reconciled, given that controlled processes are generally thought to be conscious and deliberative (i.e. system 2) (Evans and Stanovich 2013, p. 225).

Using the tripartite model of cognition proposed by Shea and Frith (2016), as well as the active inference model of flow provided by Parvizi-Wayne et al. (2024), we will endeavour to resolve this seemingly challenging dilemma, demonstrating that the intuitive aspect of flow *is* in fact that which renders it a state of cognitive control. Furthermore, we will highlight the importance of conscious representations in flow and intuition—as well as intuition in flow—insofar as they facilitate the information integration necessary for a form of adaptive behavioural control capable of unfolding in the absence of deliberative reasoning.

## Bringing systems neuroscience to bear on flow and intuition

From the perspective of systems neuroscience, both flow and intuition appear to alter functional connectivity and information integration. In previous research, we argued that both phenomena can be empirically and theoretically explained by two prominent theories, namely, active inference and dynamic systems theory (and especially the latter’s focus on the notion of metastability) (Kotler et al. 2022). In this paper, we will focus primarily on the contributions of active inference.

In brief, active inference is a corollary of the free-energy principle (FEP), which states that any thing (e.g. the cognizing organism), which persists through time, has a defining set of characteristic states (i.e. an attracting set) (Ramstead et al. 2023). Through the introduction of a statistical boundary demarcating the organism from its environment—known as a Markov blanket—the FEP states that the internal states of an agent can be described as if they are inferring external states that are hidden from the agent (by its Markov blanket) (Pearl 1988, Friston 2009, 2010, 2019, Kirchhoff et al. 2018). More specifically, internal (and active blanket) states appear to be minimizing an information theoretic term known as variational free energy (VFE), which acts as an upper bound on surprisal, Shannon self-information, or—in the long term—entropy (since entropy is the path integral of surprisal) (Shannon 1948, Friston 2013). Crucially, the minimization of VFE is formally equivalent to maximizing [a lower bound on] the evidence for the generative model entailed by the agent in question (technically the maximization of the marginal likelihood of an observation, given a generative model). This means that active inference is frequently referred to as a process of self-evidencing (Hohwy 2016). By minimizing VFE, the agent is implicitly returning to its characteristic states or attracting set. These states thus require a high probability, as it seeks evidence for its model of its world or niche (Friston 2009, 2010, 2019, Ramstead et al. 2019). Furthermore, the active inference framework has been employed in neurobiological explanations of insight and creativity (Friston et al. 2017, Priorelli and Stoianov 2023, Constant et al. 2024).

The application of the FEP to sentient behaviour generally assumes particular kinds of agents that infer their own actions. This planning as inference (Attias 2003, Botvinick and Toussaint 2012, Da Costa et al. 2020) is based upon expected free energy (EFE); namely, the free energy that would be expected under a particular course of action or plan (note that every policy or plan is appraised in terms of its EFE. In other words, EFE is an attribute of a plan, policy, action or choice). This makes an agent endowed with a temporally deep generative model particularly adaptive, because it can take actions in the present to minimize the likelihood of experiencing dyshomeostatic—or ego-dystonic—outcomes in the future. For example, I will take an umbrella with me if I notice that it is raining outside, because this will reduce the likelihood of getting wet. This anticipatory planning is frequently described in terms of allostasis (Sterling 2012, Pezzulo et al. 2015, 2018, Corcoran and Hohwy 2018). Interestingly, EFE can be expressed in terms of expected information gain and expected value, where value is simply the log probability of being in an attracting state. This provides a straightforward (Bayesian) mechanics that explains both information-seeking (epistemic) and goal-seeking (instrumental) behaviour, in terms of responding to epistemic and instrumental affordances, where *affordances* can be understood in the traditional Gibsonian manner as action possibilities latent in the environment (Gibson 1979, Friston et al. 2012, Friston 2022),

A final aspect of active (planning as) inference is the potential to acquire habits by repeatedly observing one’s own responses to cues with epistemic or instrumental affordance (Pezzulo et al. 2015, Friston et al. 2016, Maisto et al. 2019). In other words, plans or policies can be learned if they are suitably cued by any context that can be inferred. As the precision weight afforded priors over policies increases, habitual priors predominate in planning—and subsequent action selection—relative to the more deliberative policy priors, afforded by their expected free energy.

Recently, Parvizi-Wayne et al. (2024) offered an active inference account of flow to ground the phenomenal characteristics of the flow state in computational terms (e.g. neuronal dynamics as a gradient flow on the VFE). They propose that flow is a cognitive state characterized by intense focus on a task, the inhibition of deep counterfactual planning and the maximization of expected value, leading to the elimination of reflective self-awareness. This is achieved by shifting attention (cf. precision weight Parvizi-Wayne 2024a) to immediate, task-specific demands, which, in flow, fluctuate rapidly and can only be attended to, given a prerequisite level of expertise. The paper also distinguished flow from habitual (mental) actions, highlighting flow’s unique nature of deliberative yet effortless engagement in activities that demand rapid and skilful responses. In this state, the brain adopts temporally ‘shallow’ but highly precise planning focusing on immediate feedback rather than the minimization of future (expected) free energy. In the language of another theoretical framework—dynamical systems theory—in flow the brain is operating at the ‘edge of criticality’, with rapid access to its repository of learned patterns matched by an ability to use these patterns to generate real-time, temporally thin predictions about current circumstances (Hesse and Gross 2014, Tognoli and Kelso 2014, Vervaeke et al. 2018, Gross 2021, Hancock et al. 2022, Toker et al. 2022). In doing so, the flowing cognitive system exhibits a pattern of transient tendencies towards stable coordination patterns in an itinerant manner as it guides adaptive, flexible, and quick behavioural responses to a highly volatile environment.

From the perspective of active inference, intuition can be understood as the brain’s nonconscious generation of expectations about the consequences of different actions, leading to a Bayes-optimal navigation of the environment, both in its physical and mental form (Freeman 2002, Buzsáki 2006). Based on implicit knowledge and past experience, this leads to a certain action readiness via precision-weighting dynamics, as the organism ‘arrives’ at the trajectory that, if fulfilled via action, minimizes the path integral of EFE (Bowers et al. 1990, Gigerenzer 2008, Rietveld and Kiverstein 2014, Friston 2022). To foreshadow this paper’s thesis, we will suggest that in flow (and perhaps more generally), intuition constitutes a habitual mental action which, given a relevant contextual cue, optimizes certain aspects of belief updating: i.e. by increasing the precision weight of probability distributions in the generative model, such as the likelihood, transition priors, and prior preferences. These three components of a generative model are usually denoted by A, B, and C tensors, respectively.^1^ This is aligned with the proposal of Volz and Zander (2014), who refer to the type of memory content utilized in intuition as *tacitly (in)formed cue-criterion relationships* developed over the course of a lifetime (cf., Gigerenzer 2008). As Parvizi-Wayne et al. (2024) make clear, it takes *time* and *practice* to form such mental habits (intuitions).

At this juncture, it is worth distinguishing intuitions, which we take to require exposure to cues and consequent responses, from instinctual responses, which too seem habitual and yet do not require practice or learning: think, for example, of withdrawing one’s hand from a hot stove. Beyond the fact that—unlike the type of intuitions employed in canonical flow states—the (e.g. infant) agent will enact said reflex at first exposure, another difference lies in the type of action engendered by the habit. A critical aspect of the account of flow in Parvizi-Wayne et al. (2024) is that cues in flow do not provoke physical actions directly; rather, they increase the precision-weight of certain beliefs encoded in the generative model which, in turn, draw the flowing organism into physical behaviour. Instinctual responses such as reflexes, on the other hand, given their rapidity and involuntary nature, can be better understood as physical actions directly engendered by the observed cue: in this case, nociceptive input. Technically, this distinction is modelled by habitual priors (E) that have to be learned and prior preferences (C) that underwrite expected free energy. Prior preferences can be learnable but certain innate or instinctual preferences may be endowed epigenetically and manifest in the absence of experience or learning. This leads to a distinction between instinctual behaviour driven by the expected cost (C) of action (e.g. the cost of not withdrawing one’s hand from a hot stove) and intuitive (i.e. habitual) behaviour based upon (E) experience (e.g. not touching things that could be hot stoves).

Recall here that what is cultivated through experience is the association of a cue (e.g. seeing the hot stove) and a mental action which triggers high precision over prior preferences, inducing the action-readiness quintessential of intuition. Given that instinctive responses lack this intermediary mental action, it is unsurprising that the phenomenal character of action-readiness is absent in their execution. However, it is worth recognizing that, although we conceive of the presence/absence of intermediary habitual mental actions in a binary manner, thereby leaving instinctual responses in a category of their own kind, apt scepticism has been expressed by numerous researchers with respect to the type of dichotomies that our conceptual demarcation of intuition and instinct rests on (e.g. learnt versus innate; environmental versus genetic) (Johnston 1987, Oyama et al. 2003, Bateson and Gluckman 2011, Blumberg 2017). With respect to this dimension, therefore, we are disinclined to dichotomise intuition and instinct in any strong sense, recognising that, as Blumberg (2017, p. 7) puts it, ‘behaviors lie along a continuum from highly malleable or plastic to highly rigid or robust’.

## The theoretical overlap between intuition and flow—a DPT perspective

Järvilehto (2016) offers the first theoretical examination of the relationship between intuition and flow, through the lens of DPT. Dual process theory posits two separate cognitive systems: system 1 and system 2. In brief, system 1 involves fast, parallel and non-conscious information processing; system 2 involves slow, conscious and serial information processing (Tversky and Kahneman 1974, Kahneman and Tversky 1984, Sloman 1996, Stanovich and West 1998, 2008, Evans 2003, Kahneman 2011, Da Silva 2023). It is important to note that the term ‘dual process’ represents a family of theories, not a single monolith (see Evans and Stanovich 2013). For Evans (2008, 2010) and Stanovich (2005), Stanovich (2011) encourage the disuse of the labels ‘system 1’ and ‘system 2’, given the (false) indication that the two types of processes belong to just two neurological systems. For reasons that will become apparent, we will continue to speak of system 1 and system 2. In any case, it is still widely accepted that there are two distinct types (or systems) of processing, whereby system 1 refers to rapid, automatic, non-conscious processing, whereas system 2 refers to deliberative, conscious, higher-order reasoning processes (Evans and Stanovich 2013). Thus, we follow Järvilehto (2016, p. 96), who, recognizing the multitude of sub-theories belonging to DPT, concludes nevertheless that ‘System 1 and System 2 suffice as abstractions that differentiate non-conscious and conscious thought processes’.

From this perspective, Järvilehto (2016, p. 101) argues that intuition rests on ‘ontogenetic System 1 processes that have been acquired through experience and practice’. Similarly, he holds that flow is ‘a state where System 1 processes are carried out automatically, without the intervention of reflective System 2 (p. 101)’. Thus, intuition and flow are put forward as similar phenomena that both involve highly automatized nonconscious processing. In fact, Järvilehto (2016, p. 103) takes this further, proposing—as noted above—that ‘flow can be thought of as intuitive action, whereas intuition can be thought of as cognition in flow’.

While this research provides a first hypothesis about the relationship between intuition and flow, several questions remain: Does Järvilehto’s proposal align with the underlying neurobiological and neurodynamical account of these processes? Indeed, if, as we maintain, flow is a matter of cognitive control underwritten by activation of the relevant neurological regions, how would this fit with flow as intuitive action, i.e. action predicated on nonconscious information processing? Indeed, according to DPT, nonconscious, system 1 processing is not only automatic but also often leads to the wrong answer, and it is only with conscious, *controlled*, system 2 deliberative processing that individuals produce the correct solution (Kahneman 2003). With this in mind, it seems unclear how flow can be the site of expert performance *as well as* intuition, given that system 1—to which intuition putatively belongs—often yields ‘first and hasty attempts’ that need to be ‘smooth[ed] out’ by system 2 (Zander et al. 2016, p. 8; cf., Kahneman 2011, p. 44, p. 188). In other words, it could appear that intuition impairs (rather than facilitates) cognitive control and goal-seeking and, thus, should have no place in flow states, in which goal states are consistently and reiteratively fulfilled.

Furthermore, thinking about DPT more broadly, recall that one of the most important claims made by the theory is that system 1’s processing is nonconscious, whilst system 2’s processing is conscious. This brings up an interesting, yet unanswered, question: Does consciousness affect cognition? More specifically: What is the relationship between consciousness and cognitive control, and how does this relationship relate to intuition and flow? It is to these questions that we now turn.

## Consciousness and cognitive control in flow and intuition

If Järvilehto (2016) is correct, then both intuition and flow involve system 1 processes. However, recall that a growing body of evidence suggests that flow is underpinned by co-activation in both the cognitive control network and the goal-directed (reward) network (Huskey et al. 2018, 2022), which implies that flow is a product of system 2 (cf., Doyle 2017). This conflict further brings into question the role of consciousness in these processes, and, in particular, its contribution to adaptive cognitive control. In flow, performance not only diminishes when people overtly reflect on what they are doing, but flow itself is interrupted (cf., Bergamin 2017, Beilock et al. 2002, Csikszentmihalyi 2014, p. 138, Dreyfus 2007, Shepherd 2022). In other words, the fact that the physio-cognitive processes that underwrite flow are non-conscious appears to be a prerequisite for its very manifestation, aligning flow with system 1. Nevertheless, flow is not entirely non-conscious; in fact, its continuation is underpinned by the flowing individual’s very absorbed attention on the given activity’s sensory inputs and outputs. Thus, the representations over which those physio-cognitive processes occur in flow are conscious, indicating, to put it briefly, that consciousness might be good for *something* (Rosenthal 2008, Frith and Metzinger 2016, Shea and Frith 2016).

More broadly, some researchers argue that consciousness might not necessarily enhance cognition, citing evidence that well-adapted behaviours can occur without it, and that, in some situations, conscious awareness impedes optimal performance (Levine et al. 1996, Jueptner et al. 1997, Fletcher et al. 2005, Króliczak et al. 2006, McKay et al. 2015, Frith and Metzinger 2016). However, in other cases, the evidence shows that consciousness aids cognition and that when System 2 thinking is compromised by cognitive load, subjects make errors in performance (Jonides 1981, Jacoby et al. 1993, Kahneman 2003, Ericsson 2006, Schwiedrzik et al. 2011, Maciejovsky et al. 2013, Shea et al. 2014). In sum, there remains an open question as to the function of consciousness, a dilemma which appears particularly pronounced with respect to flow states, in which consciousness of the processes one is implementing to achieve flow disrupts the very experience, whilst consciousness of the sensory inputs and outputs circumventing that process is necessary for its emergence and maintenance (Gold and Ciorciari 2020).

In resolving this apparent paradox, we can start by noting that, in describing the issue at hand, we have implicitly distinguished between the non-conscious *processing* and the conscious *representations* present in flow. This distinction is informed by the work of Shea and Frith (2016, p. 1), who, in investigating ‘what is consciousness good for?’ distinguish two dimensions underlying dual process theories: representation and processing. Recognizing that the traditional DPT model only focusses on the latter, they posit a distinction between what they call ‘type 1’ and ‘type 2’ cognition with subtle yet important differences from the forerunning DPT model and the associated notions of system 1 and system 2 processing. Their notion of type 1 cognition aligns with traditional ideas about system 1 as automatic, load-insensitive fast processing. However, they characterize type 1 as operating on conscious representations, whereas system 1 is described as generally nonconscious, without regard for the phenomenal status of the relevant representations (Evans 2003, Hagger 2016, Nadurak 2021). Given this, they introduce the concept of type 0 cognition, which involves nonconscious processing over nonconscious representations. Type 2, in turn, matches system 2 as controlled, effortful processing, involving both conscious representations and conscious processing. In sum, they argue that type 0 cognition involves both non-conscious representations and non-controlled processing; type 1 consists of automatic, non-conscious processing of conscious representations; and type 2 involves both conscious representations and conscious, deliberative processing over those representations (Shea and Frith 2016).

For our purposes, Shea and Frith’s (2016) nuanced framework is useful in resolving unanswered questions. As mentioned before, the nonconscious automatic processing of type 0 and type 1 cognition enables rapid, efficient behaviours in predictable settings without requiring conscious, deliberative control; what might be termed ‘nonconscious cognitive control’. This type of offline operation is constitutive of flow, and it is this aspect which (partially) confirms Järvilehto’s (2016) claim that flow is intuition in action—that is, flow, like intuition, involves nonconscious automatic processing. Indeed, as mentioned earlier, deliberative reasoning about the underlying processes disrupts flow (Beilock et al. 2002, Bergamin 2017, Csikszentmihalyi 2014, p. 138, Dreyfus 2007, Shepherd 2022). That said, flow is not entirely nonconscious, and involves conscious representations of sensory inputs and outputs. Thus, flow is an example of type 1 cognition under Shea and Frith’s (2016) framework, coupling conscious representations with nonconscious processing.

This distinction arguably separates flow from intuition, whose representation one might imagine are nonconscious, rendering it a form of type 0 cognition. However, recall the example of one’s intuition to not go down a dark alley. This is certainly not a nonconscious process: I must consciously recognize where I am and consciously sense (as a product of my non-conscious intuitive processing) trepidation and unease, often accompanied by the covert self-talk: *don’t go in there*. From this perspective, intuition is also an example of type 1 cognition—or, if one were unwilling to accept its affective output as a representation—a type 0 (nonconscious output)/type 1 (conscious input) hybrid (Topolinski and Strack 2009). Indeed, as in flow states, individuals are not aware of the cognitive processes going on in the background—i.e. ‘they cannot report about the cues they use for making the inference nor about potential integration processes’ (Volz and Zander 2014, p. 30).

The question consequently arises as to what the function of conscious representations is in flow states and intuition. Herein lies the utility of the model proposed by Parvizi-Wayne et al. (2024). The reader will be reminded that active inference postulates a relationship between habitual tendencies (priors over policies, denoted by E) and expected free energy (denoted by G) in determining action (policy) selection. This can be formulated as:


(1) $${\mathrm{Q}}\left( {\mathrm{u}} \right)\,{\mathrm{ = }}\,{\sigma }\left( {{\mathrm{E}}\,-\,{\mathrm{G}}} \right)$$

(cf., Parvizi-Wayne and Severs 2024). Here, Q(*u*) represents the probability of an action (*u*) being selected. The equation leverages a normalized exponential function (SoftMax function: σ), which takes the vectors E and G as its arguments and converts them into a probability distribution; in this case, the likelihood of choosing a particular action (*u*). The term E represents habitual priors, which can be thought of as the probability of policies cued by specific contexts, such that, when one infers oneself to be in a given context, one would select that policy in a non-deliberative fashion (Pezzulo et al. 2015, Friston et al. 2016, Maisto et al. 2019). The term G represents the EFE, which, recall, scores how much free energy the agent expects to encounter in the future, under each policy.

In what follows, we consider action in the more general sense to include mental action, which can be regarded as selecting the most likely likelihood and transition probabilities that are most apt for inferring latent or hidden states of affairs. For example, if it is dark I may assign more precision weight to auditory input, as opposed to visual input. This is generally discussed in terms of selective attention and sensory attenuation, on a psychological review. In short, action can be covert as well as overt, which means my plans and decisions pertain to covert mental actions that can be read as action selection, in the sense of attentional selection (Feldman and Friston 2010, Limanowski and Blankenburg 2013, Limanowski and Friston 2018, Sandved-Smith et al. 2021, Limanowski 2022). One can also consider mental actions that increase the precision weight of prior preferences, which emphasize pragmatic goal-seeking during planning and, indeed, increase the precision of habitual priors that themselves prescribe mental actions. In short, any mental action will change the precision weight of posterior beliefs and, necessarily, posterior predictions that underwrite EFE, namely by altering the precision weight of priors over policies based on the planning (G), relative to habitual priors (E). In principle, it is therefore possible to have deliberative habits and a larger repertoire of implicit attentional and intentional sets.

With this difference between E and G in mind, note that Parvizi-Wayne et al. (2024) hold that flow involves multiple layers of information processing. Firstly, there is the partially nonconscious, habitual cueing (E) of mental actions that select [the precision weight of] belief updating—i.e. by increasing the precision weight of the A, B, and C tensors. This aspect of flow appears to be a hybrid of type 0 and type 1 cognition, since the flowing individual consciously represents the context (sensory input), which drives the (nonconscious) mental action, without consciously representing the output (e.g. posterior beliefs; including, beliefs about plans and sub-personal intentions)—although this arguably manifests phenomenally as an affective form of action readiness, akin to trepidation or unease in other contexts (Rietveld and Kiverstein 2014). This maps onto our previous categorization of intuition as a type 0/type 1 hybrid and suggests that this partially nonconscious mental cueing of precision weight is the intuitive aspect of flow (Volz and Zander 2014). In fact, nonconscious mental action, which is cued by context and draws the agent into action via the precision weighting dynamics it engenders, might just be the cognitive substrate of intuition.

At the same time, Parvizi-Wayne et al. (2024, p. 15) hold that flow is not entirely habitual; rather, there is the ‘activation of full active inference’ at the level of state-based and perceptual-based inference, where physical action is rooted in the optimization of beliefs about states. This is reflected in the fact that the sensorimotor inputs and outputs circumscribing physical action selection in flow are consciously represented. Again, the question concerns what consciousness (of representations) facilitates in physical action selection (Shea and Frith 2016, p. 3).

Building on work grounding consciousness in information integration (Singer 1998, Tononi 2008), Shea and Frith (2016) suggest that consciousness ‘allows representations from previously unconnected domains to be integrated for computational processing’. Such integration is particularly necessary in flow states, which are marked by the volatile (yet partially predictable) environments in which they unfold (Hohwy 2022). In facilitating the integration of information, consciousness allows flowing individuals to dynamically adjust to task contingencies: for example, combining information from the visual domain (the sight of the conductor), the tactile domain (the feel of the string on my fingers) and the auditory domain (the sound of the melody) affords the violin player adaptive control in otherwise challenging contexts. This might appear to yield a certain ‘automaticity’ (Dietrich 2004); however, this does not imply that the actual unfolding of the flow state is nonconscious (Gold and Ciorciari 2020, Shepherd 2022). As put by a rock climber in a study by Csikszentmihalyi (2000, p. 43):

*It’s like when I was talking about things becoming ‘automatic’… almost like an egoless thing in a way– somehow the right thing is done without… thinking about it or doing anything at all… It just happens… and yet you’re more concentrated*.

The last line here illustrates the type 1-ness of flow powerfully: ‘it just happens’ refers to the nonconscious automaticity of information *processing*, whereas ‘you’re more concentrated’ implies that the inputs and outputs that constitute the flow state are anything but nonconscious. In fact, they are *maximally* conscious, to afford the information integration necessary for flow to continue. This is not to say that the entire complexity of the flow-inducing context is consciously represented. Given how computationally demanding such an operation would be, it is likely that the information the integrated conscious representandum holds is reduced in flow or rendered simpler (Hillis et al. 2002, Stocker and Simoncelli 2007, Shea and Frith 2016). This might explain why flow states engender such a laser-like attentional focus, as irrelevant contextual details are pruned from consciousness.

Importantly, note that this sensory input is the same sensory input that drives the habitual mental action in flow. Whether one wishes to analyse such inputs with respect to the flow state as a whole or solely as a cue for mental action ultimately depends on the lens of analysis one adopts. In other words, consciousness, in facilitating the integration of information across domains, ensures that the contextual cue—which triggers mental actions—is the *correct* contextual cue, an example of intuition guiding robust cognitive control. *Only if* this contextual cue is appropriate can the appropriate action be selected, for the habitual mental action driven by the contextual cue sets the heightened precision-weighting that, according to active inference, is a prerequisite for the execution of the type of pragmatic action typical of flow. In this way, the apparent paradox of heightened intuition *and* cognitive control in flow mentioned above is illusory. The control constitutive of flow is a result of its intuitive nature.

That said, the outputs of the mental actions and physical actions involved in flow are different. Whereas the former is a non-conscious increase in precision-weight, the latter is a set of sensory observations contingent on the action just performed. Thus, the obvious question now pertains to the functional role of the conscious output. The answer here is simple: the represented output at time *n* just is a represented input at the same time (Hohwy 2022, Parvizi-Wayne 2024b). This, in turn, acts as a contextual cue for the action policy at *n + 1*, which will produce another sensory output/input amalgam which sets off the next bout of sensorimotor loops (Parvizi-Wayne et al. 2024). Thus, the strict distinction Shea and Frith (2016) carve between sensory inputs and outputs can be relaxed somewhat in the case of flow, in which, due to its diachronic, integrated nature, representational outputs just are representational inputs.

Note that our conception of flow as involving rolling sets of contextual cues allows us to eschew any picture of flowing agents as mechanical zombies constrained by their dispositions to a single action path (Hutto and Robertson 2020, Miyahara and Robertson 2021, Cappuccio 2023). On the contrary, the breadth of expertise invariably possessed by flowing agents opens up multiple novel and seemingly creative policies within a single overarching activity, as each particular environmental contingency acts as a new contextual cue that leads, ultimately, to a specific behaviour.

Crucially, the sensory observations that engender the relevant precision weighting dynamics need not be what were exactly expected by that agent at the prior timestep; on the contrary, as a result of the environmental volatility intrinsic to a flow state, the flow experience will likely be replete with prediction errors. However, given prerequisite experience on the part of the agent, a prediction error need not be a death knell; rather, it might simply act as the first (cueing) domino in a sequence of skilled actions that can be considered creative or improvizational, but is nevertheless still subject to the same belief dynamics which underwrite the agent’s capacity to enter into flow. As such, to conceptualise the totality of an agent’s (belief-based) embodied expertise with respect to a given activity—which typically yields flow for them—is to conceptualise a tree-like or fractal structure, with multiple divergent yet overlapping threads by which contextual cues and actions are held together. With this picture in mind, we can push back on a claim such as ‘it is of the essence of merely habitual practices that one performance is a replica of its predecessors’ (Ryle 2009, p. 30); in fact, the very habitual substrate of a practice that elicits flow licenses idiosyncratic streams of skilful responses to particular environmental circumstances, even if it is the case that one cue is invariably associated with one mental action.

Under this conception, the habits (or intuitive aspect) underlying flow are not examples of mere reflexive reactivity; they are coordinated, world-directed and typifying of the agent’s flexible responsiveness (Hutto and Robertson 2020). Furthermore, the belief architecture we have outlined here permits further flexibility at the level of the physical action, insofar as there are multiple ways to achieve the same preferred outcome, some of which may be more suitable than others in certain contexts. Thus, our account here blurs the line between habits and skills, since the former emerges as what Ryle (2009, p. 30) calls a ‘multi-track disposition’, underwriting heterogeneous, flexible and often improvizational performances in a variety of contexts (cf., Miyahara and Robertson 2021).

Finally, it is worth recognizing that, as Parvizi-Wayne et al. (2024, p. 18) write, ‘the boundary between flow and not-flow is likely highly precarious’. In other words, expert behaviour, especially that which is marked by a certain degree of novelty and exploration—as in musical improvization—may be marked by dynamic fluctuations in and out of flow (Benson 2003, Bergamin 2017). Within such non-flow moments, the habitual scheme that underpins the iterant pursuit of pragmatic affordances is absent, and the agent becomes tuned to the possibilities of epistemic exploration (and, indeed, may develop epistemic habits). In particular, the agent will engage in novelty seeking behaviour, which in active inference is associated with maximising information gain about the model parameters of a generative model, until such a policy yields an observation which acts as a contextual cue, drawing the agent back into loops of pragmatic value maximization that we have associated with the flow state. For these reasons, the account we have been sketching of the flow state as rooted in habitual mental action and the pursuit of pragmatic affordances is not out of place in more creative or spontaneous domains, in part because ‘flowing’ activity can be genuinely idiosyncratic in its concatenated form, in part because the broader context that researchers are inclined to associate with flow will likely involve moments of non-flow where genuinely exploratory behaviour is undertaken.

Note that, crucially, the cognitive control afforded by the conscious representations of the sensory inputs-outputs of flow states is not supplemented by the even *greater* degree of control that would be added if the *process* underlying flow were made conscious. This can be explained by way of the fact that conscious processing is computationally heavy and thus time-consuming, as well as susceptible to cognitive load (Kahneman 2011, p. 28, Shea and Frith 2016). In volatile, complex environments such as those that elicit flow—in which the individual’s planning horizon is constricted and their attention is deployed wholesale on the unfolding task dynamics—there is simply not the time nor the cognitive bandwidth to deliberatively attend to the underlying process; not, at least, if flow is to continue (Gigerenzer and Sturm 2012). Consequently, it is not so much that type 2 cognition would be a useful addendum to flow; in fact, its very nature makes it anathema to flow and its defining characteristics. What’s more, flow serves to demonstrate that expertise need not rest on deliberative reasoning, which, although necessary in the early stages of the individual’s skill acquisition, gives way to implicit, procedural knowledge and intuition-as-habitual-mental-action (Parvizi-Wayne et al. 2024). Given the necessity of conscious representations for its achievement, this is not to discount the importance of consciousness in flow (or more broadly in cognitive life); rather, it highlights that consciousness, especially when disposed onto physio-cognitive processes, brings with it certain negative externalities that might otherwise inhibit a phenomenal state like flow.

## Conclusion

In summary, this paper addresses the possible relationship between intuition and flow, positing that these two cognitive phenomena share a common ground in nonconscious processing. Furthermore, we have explained, using the formalisms of active inference and Shea and Frith’s (2016) tripartite framework of cognition, how, as Järvilehto (2016) expresses, flow is intuition in action. More specifically, we have proposed that the intuitive aspect of flow (and perhaps life more broadly) can be couched in the habitual mental action that selects the most likely (likelihood and prior) contingencies in a given context. This renders flow the zenith of performance and cognitive control without the encumbrance of conscious deliberation, albeit not without the important contribution of conscious representations.

Nevertheless, while our framework emphasizes the adaptive function of intuition and flow, it is important to recognize that these cognitive states can be suboptimal or maladaptive in certain circumstances. Intuitions, and related heuristics [e.g. Eureka Heuristics (Laukkonen et al. 2023)], can occasionally lead to suboptimal inference. In the technical literature, this is clearly evident in reversal learning paradigms (Frank 2005, Peterson et al. 2009, Lloyd and Leslie 2013, Friston et al. 2016). In this context, habits are formed on the basis of what the agent sees herself doing repeatedly, in a given context. However, if habitual priors become overly precise—and the context changes—there is a failure to engage epistemic foraging (e.g. novelty seeking) because habitual priors (E) predominate over expected free energy (G). This leads to impoverished reversal learning and a pernicious failure of context sensitivity. The same phenomena can also be observed in the setting of insights: i.e. ‘aha moments’ and ensuing ‘Eureka’ heuristics that can be modelled in terms of structure learning (a.k.a., Bayesian model selection). For example, one might commit to certain prior beliefs about precise likelihood mappings prematurely; effectively, ‘jumping to conclusions’ (Averbeck et al. 2011, Moutoussis et al. 2011); see (Friston et al. 2017) for a numerical example. Similarly, the heightened confidence associated with flow states could, albeit occasionally, result in overconfidence.^2^However, it is crucial to note that such unfavourable outcomes are exceptions rather than the rule, particularly given the well-documented benefits of flow on performance and wellbeing (Kotler et al. 2022).

Indeed, by understanding the phenomenal–computational structures and processes underwriting flow and intuition, we can appreciate the elegance of the mind’s ability to both ‘think without thinking’ and perform optimally without deliberative decision-making. This synergy between intuition and flow not only enriches our comprehension of human cognition, but also sparks a myriad of questions for further exploration. Future research, grounded in the nuanced understandings proposed by Shea and Frith (2016), will undoubtedly continue to unravel the subtle complexities of these phenomena, enhancing our understanding of how we can harness flow and intuition to elevate our lives and our performance to new levels.

Finally, it is worth recognising that the model used by Shea and Frith (2016) is explicitly computational and algorithmic, relying on notions of inputs, outputs, operations and representations, the latter construct putatively defining consciousness *per se* as well as cognition more broadly. In this rather brief treatment, we cannot attempt to do justice to the enormity of the historical debate concerning the viability of this account in explaining embodied mental activity; rather, in recognizing the ongoing tensions between different, invested philosophical parties, we simply encourage the integration of the claims made here into a nonalgorithmic, embodied, enactivist account. Crucially, such expositions should not discount the central point articulated in this paper—namely, that consciousness is neither an unequivocal good nor an unequivocal bad in (intuitive) flow, nor in our coping with the world more broadly. This can be the case even if conscious states are not held to be representations over which computational processes operate.

## Data Availability

The original contributions presented in the study are included in the article/supplementary material. Further inquiries can be directed to the corresponding author.

## Footnotes

1 Note that here we are using the language of partially observable Markov decision process (POMDP) schema used to describe active inference in discrete state space (cf., Friston et al. 2017, Da Costa et al. 2020).

2 One of us, S.K., has written about this extensively in his book The Rise of Superman: Decoding the Science of Ultimate Human Performance.
